# Peer Review of “COVID-19 and Cybersecurity: Finally, an Opportunity to Disrupt?”

**DOI:** 10.2196/29414

**Published:** 2021-05-06

**Keywords:** COVID-19, cybersecurity, challenges and disruption, data protection, privacy, health data

*This is a peer-review report submitted for the paper "COVID-19 and Cybersecurity: Finally, an Opportunity to Disrupt?"*

## Round 1 Review:

### Minor Comments

Dear Authors, the article [1] is well written; however, in my opinion, there is a need to rephrase the *Conclusion* section in order to make it more clearly informative.
